# Common Variants of *Drosophila melanogaster* Cyp6d2 Cause Camptothecin Sensitivity and Synergize With Loss of Brca2

**DOI:** 10.1534/g3.112.003996

**Published:** 2013-01-01

**Authors:** Adam M. Thomas, Carrie Hui, Adam South, Mitch McVey

**Affiliations:** * Tufts University, Department of Biology, Medford, Massachusetts 02155, and; † Tufts Sackler School of Graduate Biomedical Sciences, Program in Genetics, Boston, Massachusetts 02111

**Keywords:** cytochrome p450, homologous recombination, double-strand breaks, chemotherapeutics

## Abstract

Many chemotherapeutic agents selectively target rapidly dividing cells, including cancer cells, by causing DNA damage that leads to genome instability and cell death. We used *Drosophila melanogaster* to study how mutations in key DNA repair genes affect an organism’s response to chemotherapeutic drugs. In this study, we focused on camptothecin and its derivatives, topotecan and irinotecan, which are type I topoisomerase inhibitors that create DNA double-strand breaks in rapidly dividing cells. Here, we describe two polymorphisms in Drosophila *Cyp6d2* that result in extreme sensitivity to camptothecin but not topotecan or irinotecan. We confirmed that the sensitivity was due to mutations in *Cyp6d2* by rescuing the defect with a wild-type copy of *Cyp6d2*. In addition, we showed that combining a *cyp6d2* mutation with mutations in Drosophila *brca2* results in extreme sensitivity to camptothecin. Given the frequency of the *Cyp6d2* polymorphisms in publcly available Drosophila stocks, our study demonstrates the need for caution when interpreting results from drug sensitivity screens in Drosophila and other model organisms. Furthermore, our findings illustrate how genetic background effects can be important when determining the efficacy of chemotherapeutic agents in various DNA repair mutants.

The complexities of human genetic disorders often require model systems to provide a better understanding of the disease mechanism. *Drosophila melanogaster* provides an excellent system for human disease research because of its genetic tractability and the presence of many homologs of human disease genes in the fly genome (Rubin 2000). As such, it has been used to the study the genetic mechanisms of cancer for nearly 40 years (Gateff and Schneiderman 1974; Gateff 1978), and multiple facets of carcinogenesis have been investigated in that time (reviewed in Rudrapatna *et al.* 2012). Drosophila also has proven to be an invaluable tool to research the effects of chemotherapeutic drugs (Boyd and Setlow 1976; Radcliffe *et al.* 2002; Jaklevic *et al.* 2006; Edwards *et al.* 2011; Gladstone and Su 2011) and the effects of mutations in key DNA repair genes (reviewed in Su 2011).

We have used mutagenic chemicals and radiation to better understand the functions of critical DNA repair proteins (Chan *et al.* 2010; Kane *et al.* 2012). Of particular interest to us is the topoisomerase I (Top1) poison, camptothecin, from which the chemotherapeutic drugs topotecan and irinotecan are derived (reviewed in Legarza and Yang 2006; Pommier 2006). Camptothecin and its derivatives stabilize the normally transient covalent link between DNA and Top1, thereby interfering with the relaxation of supercoiling that occurs during events requiring DNA unwinding, such as replication or transcription (Hsiang *et al.* 1985; Hsiang and Liu 1988; Koster *et al.* 2007). The classical model proposes that single-strand breaks at the sites of camptothecin-induced Top1-DNA links are converted into double-strand breaks (DSBs) after collision with a replication fork (Holm *et al.* 1989; Hsiang *et al.* 1989). Recent research has challenged this theory, however, proposing instead that the accumulation of regressed forks or supercoiled DNA is responsible for the toxic effects (Ray Chaudhuri *et al.* 2012).

Although cancer frequently involves defects in multiple genes or pathways, there are specific examples in which mutations in a single gene are associated with significant cancer risk. Perhaps one of the most well-known examples is the breast cancer susceptibility gene, *brca2*. Individuals inheriting a mutant copy of the gene exhibit a significant increase in breast and ovarian cancer risk (Wooster *et al.* 1995). The Brca2 protein functions in homologous recombination (HR) repair of DNA DSBs (reviewed in Thorslund and West 2007 and Boulton 2006). HR uses an intact DNA template to synthesize nucleotides lost on a broken homologous chromosome or sister chromatid. It is mediated by the recombinase, Rad51 (Sung 1994), which is recruited to the sites of DSBs by Brca2 (Davies *et al.* 2001). Similar to its mammalian homolog, Drosophila Brca2 interacts with DmRad51 (Brough *et al.* 2008) and plays a critical role in both mitotic and meiotic HR repair of DSBs *in vivo* (Klovstad *et al.* 2008).

We were interested in examining the role of Drosophila Brca2 in the repair of camptothecin-induced DNA damage. To do this, we treated two stocks of *brca2* mutant flies with camptothecin. We were surprised to discover that one line of *brca2* mutants was exceptionally sensitive to the drug. Further investigation revealed that these flies carried a second mutation in *Cyp6d2*, a cytochrome P450 gene, which, when combined with the *brca2* mutation, resulted in synergistic hypersensitivity to camptothecin. We now report that many publicly available Drosophila stocks carry this mutation or a second, independent mutation in *Cyp6d2* that also causes extreme sensitivity to camptothecin.

## Materials and Methods

### Fly culture conditions and stocks

Flies were kept at 25° with an alternating 12-hr light:12-hr dark cycle and fed a standard cornmeal agar diet. Fly stocks were acquired from the Bloomington Stock Center, with the exception of *brca2*^KO^ (see below, *Creation and isolation of mutants*). For our mapping and sequencing studies, we used the sequence available on Flybase as our wild-type standard (Adams *et al.* 2000).

### Creation and isolation of mutants

The *brca2*^47^ mutation was created via an imprecise excision of *P{SUPor-P}KG02287*, located between *brca2* and *CG3746*. The excision resulted in a deletion removing the first 2169 bp of *brca2*. The *brca2*^KO^ mutant was a donation from Trudi Schüpbach’s laboratory and was created by ends-out HR (Klovstad *et al.* 2008).

The original *scpt* mutant was found in the same stock used to create *brca2*^47^ but was created via a precise excision of the *P*-element. This mutant was later renamed *cyp6d2*^SD^. The other *Cyp6d2* mutation, *cyp6d2*^NT^, was found in the *P{GT1}CG42565*^BG02301^ stock.

### Sensitivity assays

Sensitivity assays were set up using five to eight virgin female flies and three male flies. The females were heterozygous for the mutation of interest, whereas males were either heterozygous or homozygous. Heterozygous flies were balanced by the *CyO* chromosome.

Parental flies were kept in vials for 3 d to lay eggs and were then transferred to new vials. The flies were then discarded after 2-3 additional days. Each set of vials was treated with mutagen or vehicle one day after the parents were removed. Camptothecin (Sigma-Aldrich) was dissolved in DMSO and then diluted in Tween 20/EtOH (5% ethanol, 1% Tween 20). Mechlorethamine, methyl methanesulfonate, hydroxyurea (Sigma-Aldrich) topotecan, irinotecan, and bleomycin (Enzo Life Sciences) were dissolved in water. Each sensitivity trial consisted of five to eight vials. Heterozygous and homozygous offspring were counted periodically until 18−20 d after the crosses were established. Percent survival was calculated by the following equation:

$$\text{Relative}\;\text{percent}\;\text{survival}=\frac{\text{percent}\;\text{of}\;\text{homozygous}\;\text{flies}\;\text{in}\;\text{treated}\;\text{vials}}{\text{percent}\;\text{of}\;\text{homozygous}\;\text{flies}\;\text{in}\;\text{control}\;\text{vials}}\times 100\%$$

Sensitivity to ionizing radiation was characterized in a similar way. Parental flies (40−60 virgin females and 10−20 males) were allowed to lay eggs on grape agar plates for several days, with plates replaced every 10−14 hr. The plates were then supplemented with yeast paste and placed at 25°. Once larvae reached third instar stage, the grape agar plates were irradiated at a rate of 800 rads/min in a Gammator 1000 irradiator. After irradiation, the larvae were moved into fly bottles for further development. Adult flies were counted and sorted as described previously, and irradiated flies were compared with an unirradiated control.

Rescue sensitivity assays consisted of five to eight virgin female flies that were heterozygous for the mutation of interest (balanced by *CyO*) as well as one copy of the *Cyp6d2* rescue construct (see below, *Construction of the Cyp6d2 rescue stock*). They were crossed to three males that were heterozygous or homozygous for the mutation tested, with no rescue construct. Vials were treated as described previously, and the progeny were sorted by wing type and eye color. Percent survival was calculated as described previously but separately for rescued and nonrescued flies.

### Mapping

For complementation mapping, flies heterozygous for a defined deletion or insertion (over 2nd chromosome balancer *CyO*) were crossed to *scpt* homozygous flies in vials. Vials were treated as described previously for sensitivity assays, using the *scpt* lethal dose of 25 μM camptothecin. Flies were sorted by wing type to determine complementation.

Meiotic mapping used noncomplementing stocks carrying *white*^+^ markers at defined locations. Female flies heterozygous for the noncomplementing marked deletion or insertion (over a wild type 2nd chromosome to allow for meiotic recombination) were crossed to homozygous *scpt* mutant males in vials. Vials were treated with 25 μM camptothecin. Offspring were sorted by eye color, and the percent of red-eyed progeny was calculated.

### Construction of the Cyp6d2 rescue stock

The full-length *Cyp6d2* gene, including 402 bp upstream of the transcription start site and 285 bp downstream of the transcription termination site, was amplified by polymerase chain reaction (PCR) using Phusion polymerase (New England Biolabs) and the primers 5′-TCTAGAGGTACCGCGCTGACAATCCTACAAGC-3′ and 5′-AGATCTGCGGCCGCGATTCCGCAAGGTGGAGAAG-3′. The PCR product was digested with *Acc*65I and *Not*I and directionally cloned into *pattB* (gift of K. Basler). Purified plasmid (500 ng/μL) was injected into fly embryos <2 hr after egg laying containing an *attP* site on 3R: *y*^1^,M{vas-int}ZH2A,w*; *M{3xP3-RFP,attP}ZH96E*. Injected embryos were allowed to develop into adult flies at 25°. These adults were crossed to *w*^1118^ males or females, and the progeny sorted by eye color. We recovered at least one red-eyed fly in the progeny of approximately 6% of all surviving injected flies. The presence of the construct was confirmed by PCR and sequencing. Flies carrying the rescue construct were mated to mutant flies to generate stocks carrying both the mutation of interest and the rescue construct. These flies were used in rescue sensitivity assays as described above.

### Allele-specific PCR

To identify stocks with specific *Cyp6d2* mutations, allele-specific primers were each paired with the genomic primer downstream of *Cyp6d2*, 5′-ctctcgaattcagaacgagc-3′. The allele-specific primer, 5′-GGGTCCTAGGCACTGCAGAC-3′, was used to detect *cyp6d2*^SD^, with an annealing temperature of 60°. The allele-specific primer, 5′-CCCATCGCTTCGATTCAGAGAC-3′, was used to detect *cyp6d2*^NT^, with an annealing temperature of 56°. These pairs yield 391-bp and 452-bp products, respectively, when we amplified template DNA from the appropriate mutant but produce no product with a wild-type template. Phusion polymerase (New England Biolabs) was used for amplification.

### Reverse-transcriptase PCR

To determine expression levels of *Cyp6d2* in various backgrounds, 15 wandering 3rd instar larvae (5−6 d after egg laying) were isolated and frozen at −80°. RNA was purified using RNAqueous-4PCR (Ambion) and cDNA was synthesized using RETROscript (Ambion). Random decamers were used as primers for cDNA synthesis. *Cyp6d2* cDNA was then amplified to detect transcripts in each genetic background. The following primers were used: XInt1 5′-CATTAGCTTAGCAATCGGTGG-3′; XInt2 5′-GGACATCTGCATCATGGAAACC-3′; XInt3 5′-CTTTTCCCATGCGAAGAGCTATGC-3′; F1 5′-CTCGCCAAATCATGACCAGC-3′; F2 5′-GCTAAGCTAATGAACCGCTTGG-3′; R1 5′-GCGGCATCGAAACGGAACTC-3′; rp49F 5′-CCATCCGCCCAGCATACAGG-3′; and rp49R 5′-CTCGTTCTCTTGAGAACGCAG-3′.

## Results

### Identification of a genetic modifier of camptothecin sensitivity

Drosophila Brca2 has been shown to play a critical role in HR repair of DNA DSBs (Brough *et al.* 2008; Klovstad *et al.* 2008). Along with its clinically approved analogs topotecan and irinotecan, camptothecin is traditionally thought to create DSBs in rapidly dividing cells via replication run-off (Strumberg *et al.* 2000), leaving a one-ended DSB (Tsao *et al.* 1993). Brca2-dependent HR repair could then be required to restart replication.

**Figure 1:**
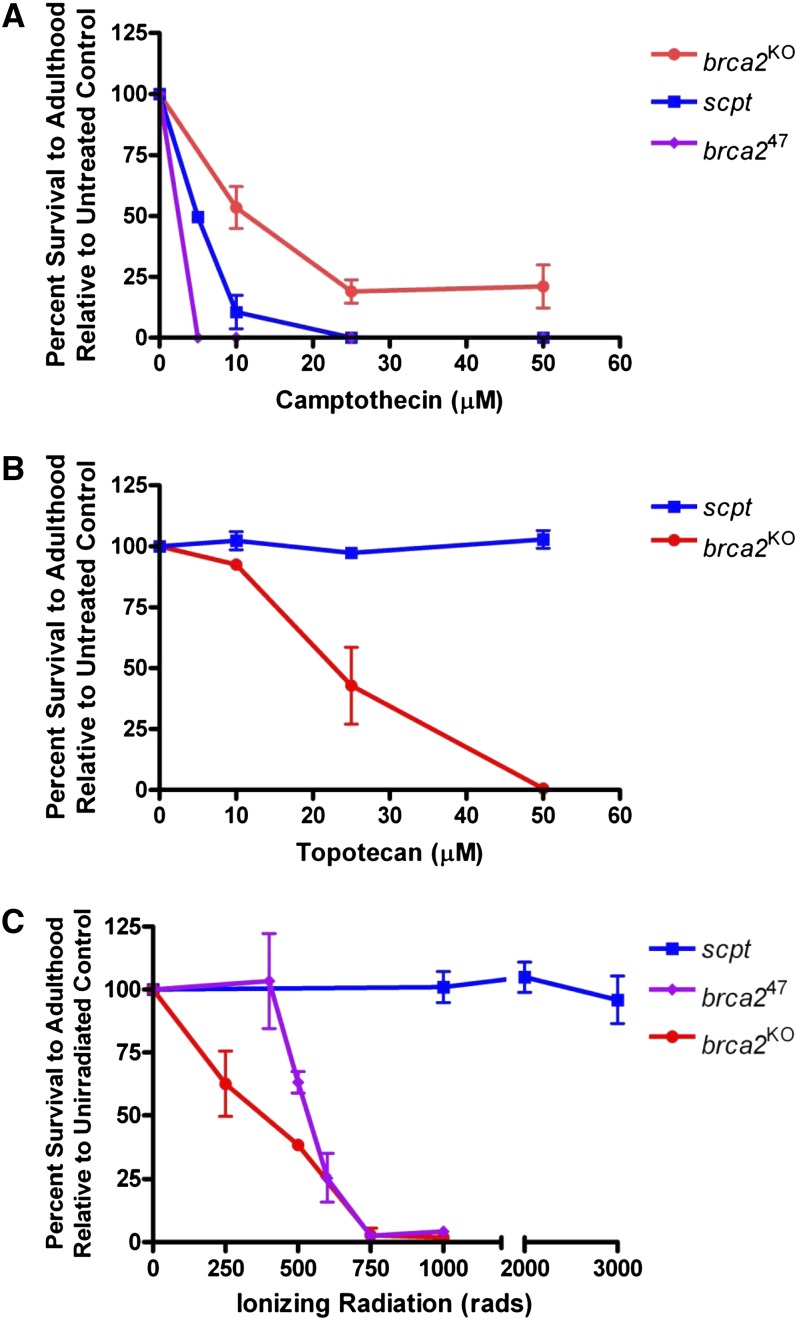
The *scpt* mutant is sensitive to camptothecin but not to topotecan or ionizing radiation. (A) Camptothecin sensitivity of the *scpt* mutant as well as two null alleles of *brca2*. All data points represent three independent trials of five to six vials each. (B) Topotecan sensitivity of the *scpt* mutant and *brca2*^KO^ mutant. All data points represent three independent trials of five to six vials each. (C) Ionizing radiation sensitivity of the *scpt* mutant as well as two *brca2* mutant alleles. All data points represent two to four independent trials of one bottle each. SDs are shown as error bars.

To test this hypothesis, we treated two independently derived *brca2* mutants with camptothecin and quantified survival to adulthood. The first was a null allele (*brca2*^KO^) created by ends-out HR (Klovstad *et al.* 2008). The second (*brca2*^47^) was created via an imprecise excision of a *P*-element 90 bp upstream of *brca2* (*P{SUPor-P}KG02287*) and deletes the 5′ half of *brca2*. Both alleles were sensitive to camptothecin, suggesting that Brca2-mediated HR repair is important for repair of one-ended DSBs. Surprisingly, the *brca2*^47^ mutant was significantly more sensitive to camptothecin than the *brca2*^KO^ allele (Figure 1A). We suspected that the *brca2*^47^ stock contained a second-site mutation that made it more sensitive than the *brca2*^KO^ mutation. Such mutations are common in imprecise excision screens, and often occur near the site of the inserted element.

To confirm this, we created a precise excision of *P{SUPor-P}KG02287*. Sequencing of DNA from this stock revealed that the *brca2* coding sequence was identical to wild-type sequence. Surprisingly, this precise excision stock also displayed high sensitivity to camptothecin, although not to the level of *brca2*^47^ flies (Figure 1A). This suggested that the second-site mutation did not arise during the imprecise excision of the *P*-element but rather was present in the original stock used to create *brca2*^47^. We named this mutation *scpt* (sensitive to camptothecin). Unlike *brca2* mutants, *scpt* mutants were not sensitive to the camptothecin analog topotecan (Figure 1B) or to ionizing radiation (Figure 1C). Furthermore, *scpt* mutants were not sensitive to irinotecan, bleomycin, mechlorethamine (nitrogen mustard), methyl methanesulfonate, or hydroxyurea (data not shown).

### The *scpt* mutation is present in multiple stocks

To understand the unusual specificity of the *scpt* mutation, we sought to identify the gene(s) mutated in this stock. Since *scpt* exhibited Mendelian segregation with the 2nd chromosome balancer *CyO*, we assumed the mutation was located on the 2nd chromosome. To map the mutation, we began by performing complementation tests using chromosome *2* deficiency stocks. We crossed deficiency stocks to *scpt* mutants and treated the offspring with 25 μM camptothecin, a lethal dose for *scpt* homozygotes. Our initial tests used the DrosDel (ED) Deficiency Collection (Ryder *et al.* 2004), available from the Bloomington Stock Center.

We were surprised to find that none of the 2nd chromosome fly stocks we used from this collection were able to complement the *scpt* mutation when treated with a lethal dose of camptothecin, regardless of the location of the deletion (Table 1). However, multiple stocks from the Exelixis and Bloomington Stock Center deficiency collections did complement the mutation at this dose. Therefore, we conclude that the progenitor stocks of the DrosDel collection as well as *P{SUPor-P}KG02287* carry the *scpt* mutation.

**Table 1:** Cyp6d2 status of various stocks

Stock*^a^*	Cytological Location	*Cyp6d2* Allele*^b^*	Complementation Test*^c^*
*P{SUPor-P}* insertions			
KG00490 (*CG34370*)	58B1	WT	Complements
KG01596 (*whd*)	47A11	WT	Complements
KG04872 (*CG13322*)	49E1	WT	Complements
KG06046	60F5	WT	Complements
KG06805 (RabX1)	59E2	WT	ND
KG07568 (*CG15704*)	53A4	WT	Complements
KG00006 (*CycB*)	59B2	SD	Does not complement
KG02089 (*Hrb87F*)	87F7	SD	ND
KG02287 (*CG4612*, *brca2*)	60D4	SD	Does not complement
KG02463 (gce, *Top1*)	13B6	SD	ND
KG02566 (*CG10880*)	40F1	SD	Does not complement
KG05061 (*Babos*)	58D4	SD	Does not complement
KG06675 (*CG9896*)	59C1	SD	Does not complement
KG07633 (*Egfr*, *CG30286*)	57E9	SD	Does not complement
KG07401 (*CG13511*)	58F4	SD	Does not complement
KG07430 (*Tim17b2*)	35D2	SD	Does not complement
KG06763	35B1	SD	Does not complement
KG07930 (*Jheh3*)	55F8	SD	Does not complement
*Mi{MIC}* insertions			
MI02105 (*grp*)	36A10	NT	ND
MI02462 (*Mad*)	23D3	NT	ND
MI00056 (*jbug*)	59A3	WT	ND
MI02085 (*Cyp6d2*)	58F4	WT	ND
*Mi{ET1}* insertions			
MB00453 (*dp*)	25A1	NT	ND
MB01292 (*CG3746*)	58F4	NT	Does not complement
MB05269 (*Cyp6d2*)	58F4	NT	Does not complement
MB05513 (*CG13579*)	60D1	NT	ND
*P{GT1}* insertions			
BG02301 (*CG42565*)	58F4	NT	Does not complement
BG02743 (*Hrb87F*)	87F7	NT	ND
DrosDel deficiencies			
Df(2L)ED284	25F2-26A3	NT	Does not complement
Df(2L)ED1303	37E5-38C6	NT	Does not complement
Df(2R)ED3683	55C2-56C4	NT	Does not complement
Df(2R)ED3728	56D10-56E2	NT	Does not complement
Df(2R)ED3923	57F6-57F10	NT	Does not complement
Df(2R)ED3952	58B10-58E5	NT	Does not complement
Df(2R)ED4061	60C8-60D13	NT	Does not complement
Df(2R)ED4065	60C8-60E8	NT	Does not complement
Df(2R)ED4071	60C8-60E8	NT	Does not complement
Df(3R)ED4408	66A22-66C5	NT	ND
Df(3R)ED10257	83A7-83B4	NT	ND
Bloomington Stock Center deficiencies			
Df(2R)BSC597	58A2-58F1	WT	Complements
Df(2R)BSC598 (*Cyp6d2*)	58F3-59A1	Deleted	Does not complement
Df(2R)BSC599	59B1-60F5	WT	Complements
Df(2R)BSC602	60C8-60E5	WT	Complements
Df(2R)BSC603	60C7-60D1	ND	Complements
Df(2R)BSC604	60D4-60E11	ND	Complements
Df(2R)BSC605	60D8-60E8	ND	Complements
Df(2R)BSC606	60D10-60E1	WT	Complements
Df(2R)BSC607	60E4-60E8	WT	Complements
Df(2R)BSC769	59B7-59D9	WT	Complements
Df(2R)BSC784	59B4-59B6	ND	Complements
Df(2R)BSC787	58F4-59B1	ND	Complements
Exelixis deficiencies			
Df(2R)Exel6044	37F2-38E3	ND	Complements
Df(2R)Exel6079	59A3-59B1	WT	Complements
Df(3R)Exel6178	90F4-91A5	WT	ND

ND, no data; WT, wild type.

a Genes potentially affected by transposon insertions are shown.

b WT: sequence matches Flybase at two loci of interest; SD: stock carries *cyp6d2*^SD^ allele; NT: stock carries *cyp6d2*^NT^ allele.

c Stock tested for complementation (survival) with original *scpt* allele (precise excision of *P{SUPorP}KG02287*) at a dose of 25 μM camptothecin.

Because multiple DrosDel stocks were carriers, we wanted to see whether other chromosomes containing *P{SUPor-P}* elements also carried *scpt*. We performed complementation tests as before, using *P{SUPor-P}* stocks. Roughly two-thirds of the *P{SUPor-P}* stocks tested did not complement the *scpt* mutation (Table 1). Location of the *P{SUPor-P}* element did not correlate with complementation of *scpt*. Thus, we hypothesize that the progenitor stock used to create the *P{SUPor-P}* collection (Roseman *et al.* 1995) carried the mutation, although it appears to have been lost in a subset of these stocks.

### Mapping the *scpt* mutation

**Figure 2:**
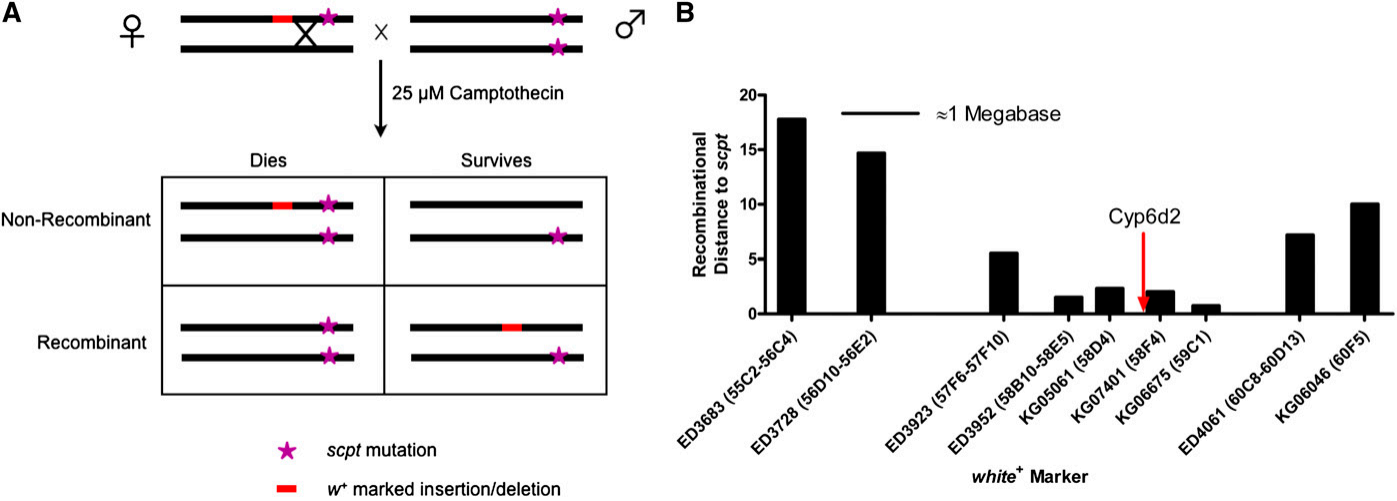
Meiotic mapping of the *scpt* mutation. (A) Cross scheme for meiotic mapping. Flies that inherit the *white^+^* marker have red eyes. Flies that inherit two mutant copies of the *scpt* mutation do not survive treatment with 25 µM camptothecin. The percentage of surviving flies with red eyes should be equal to the recombination distance between the *white*^+^ marker and *scpt*. (B) Multiple meiotic mapping crosses identified a region containing the *scpt* mutation. Both DrosDel deficiencies (ED) and *P{SUPor-P}* elements (KG) were used. Distances on the x-axis are approximations of the physical distance between these markers. The region shown spans from cytological band 55C to 60F (the terminal quarter of chromosome 2R, or about 7 megabases).

Because the DrosDel stocks and many *P{SUPor-P}* stocks carry the *scpt* mutation at an unknown location and a red eye marker (*white^+^*) at a defined location, we used traditional meiotic mapping to determine the location of *scpt* (Figure 2A). Unbalanced female flies heterozygous for the *scpt*, *white^+^* chromosome (from either *P{SUPor-P}* or DrosDel stocks) were crossed to white-eyed homozygous *scpt* males, and the progeny were treated with 25 μM camptothecin. Lower-than-expected survival rates of red-eyed flies (<50%) indicated linkage of the *white^+^* marker to *scpt*. We did this using multiple DrosDel and *P{SUPor-P}* stocks near the right end of chromosome 2 (Figure 2B). These tests indicated a location of interest near the cytological bands 58B−59C.

We further refined the position of *scpt* with complementation tests using deficiencies from the Exelixis and Bloomington Stock Center collections. Based on our complementation tests (Table 1), we assumed that these deficiency collections did not carry *scpt*, and therefore noncomplementation indicated that the deficiency deleted the gene(s) mutated in *scpt* flies. Of the five deficiencies near the region of lowest recombination distance, only *Df(2R)BSC598* did not complement the *scpt* mutation. This suggested that *scpt* was located in the 36.9-kb region deleted in this deficiency. Furthermore, we found that an overlapping deletion in *Df(2R)BSC787* complemented *scpt*, allowing us to narrow down the *scpt*-containing region to approximately 20 kb. This region contains 13 genes, one of which is the cytochrome P450 gene, *Cyp6d2*.

We sequenced the coding regions of all 13 of these genes using flies from complementing *P{SUPor-P}* stocks (*KG06046* and *KG01596*) and noncomplementing *P{SUPor-P}* stocks (*KG02287* and *KG06675*). Amino acid changes we identified in the noncomplementing stocks are shown in supporting information, Table S1. The complementing *P{SUPor-P}* stocks matched the published *Drosophila* sequence found on FlyBase at almost all locations. Importantly, the Flybase sequenced stock, *y^1^*; *Gr22b^1^ Gr22d^1^ cn^1^ CG33964^R4.2^ bw^1^ sp^1^*; *LysC^1^ MstProx^1^ GstD5^1^ Rh6^1^* (Adams *et al.* 2000) was not sensitive to camptothecin (data not shown).

### The *scpt* mutation alters splicing of the Cyp6d2 transcript

**Figure 3:**
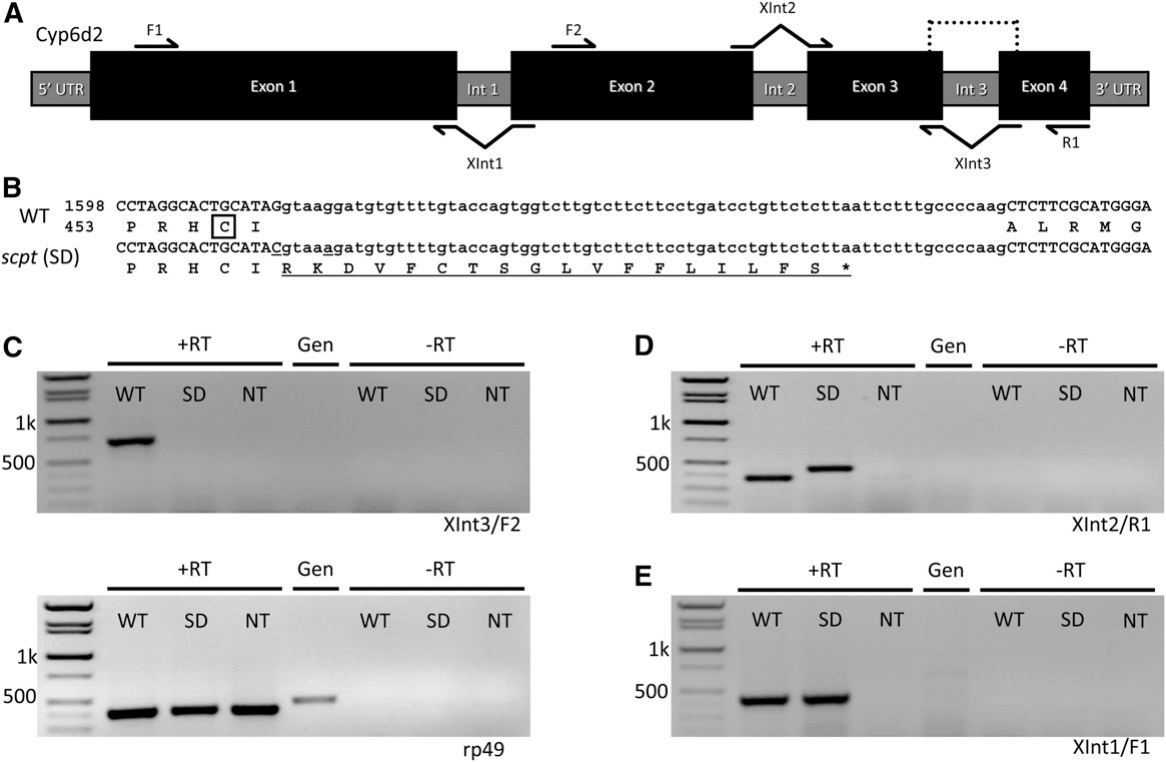
Mutations in *Cyp6d2* affect transcription and RNA processing. (A) Diagram of the *Cyp6d2* gene structure. Protein coding regions are represented by black boxes, whereas gray bars represent introns and UTRs. Dotted line indicates the region shown in B. Primers shown were used in RT-PCRs in C−E. (B) Nucleotide and amino acid alignment of wild-type (WT) and *scpt* mutant Cyp6d2. *scpt* SNPs in this region are underlined. The heme-binding cysteine is boxed in the WT protein. Failure to splice intron three (sequence in lower-case letters) results in a frameshift mutation and a premature stop codon (*). Total wild-type protein length is 512 amino acids. (C−E) Semiquantitative RT-PCR of wild-type and mutant larvae. Primers indicated below gels refer to those shown in (A). RT-PCR with (+RT) and without (−RT) reverse transcriptase was performed. Genomic (Gen) DNA template is also shown for comparison. Sizes of molecular weight markers are indicated. (C) The XInt3/F2 primer pair yields a PCR product of 709 bp if splicing of intron 3 is correct. The rp49 primer pair (control reaction) yields PCR products of 398 bp and a genomic product of 460 bp. (D) The XInt2/R1 primer pair yields a PCR product of 375 bp if introns 2 and 3 are properly spliced. Intron 3 length is 70 bp. (E) XInt1/F1 yields a PCR product of 440 bp if intron 1 is correctly spliced.

Two of the polymorphisms detected by sequencing were located near the exon three/intron three junction in *Cyp6d2* (Figure 3, A and B). The first (G→C) is located at the terminal nucleotide of exon three and is also the first nucleotide of an alanine codon. The second (G→A) is located five nucleotides downstream of the exon−intron junction. If the two single-nucleotide polymorphisms (SNPs) did not affect splicing, the first SNP would change the alanine (A458) to a proline. Alignment of *Drosophila* Cyp6d2 and other P450 enzymes indicated that this change is in a highly conserved region identified as the heme-binding domain (Tijet *et al.* 2001). Alternatively, if the two SNPs combined to disrupt splicing of intron three, the alanine would become an arginine and a frameshift would result in a premature stop codon 17 amino acids downstream.

To distinguish between these two possibilities, we performed semiquantitative reverse transcription (RT)-PCR to analyze *Cyp6d2* transcripts in wild-type and *scpt* mutant larvae. We synthesized total cDNA from RNA purifications and amplified DNA using primers that will anneal only to correctly spliced exons (Figure 3A). The pairing of a reverse primer that spans intron three with a forward primer in exon two resulted in no product for *scpt* mutant larvae, whereas both the *scpt* mutant and wild-type larvae had normal levels of the *rp49* control transcript (Figure 3C). In addition, pairing a forward primer spanning intron two with a reverse primer in exon four produced a larger than expected product for *scpt* larvae (Figure 3D). These results are consistent with a splicing defect in *scpt* mutants and suggest that the intron three sequence is included with the final transcript. Sequencing confirmed that the cDNA templates isolated from the *scpt* mutant included intron three (data not shown). To demonstrate that splicing of other *Cyp6d2* introns is normal in the *scpt* mutant, we paired a reverse primer that spans intron one with a forward primer in exon one. This produced equal length products for wild-type and *scpt* mutant larvae (Figure 3E). Based on these findings, we reasoned that the *cyp6d2*^SD^ (splicing defective) allele was the best candidate for the *scpt* mutation.

To confirm that *cyp6d2*^SD^ was the *scpt* mutation, we sought to rescue the camptothecin sensitivity with a wild-type copy of *Cyp6d2* by using phiC31-mediated transgenesis (Bischof *et al.* 2007). To do this, we cloned and inserted wild-type *Cyp6d2*, along with several hundred base pairs of flanking sequence, into the vector *pattB*. The *pattB-Cyp6d2* plasmid was injected into fly embryos carrying an *attP* site at cytological band 96E. Successful germline integration of the plasmid was detected in the following generation by screening for *white^+^* flies.

**Figure 4:**
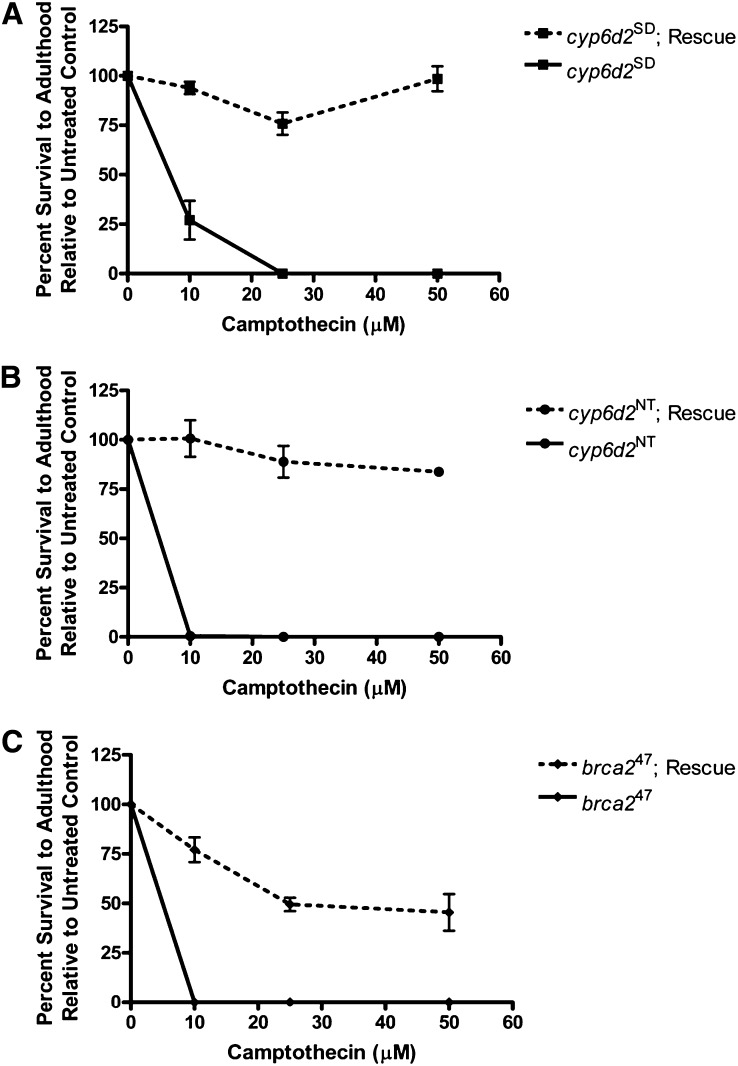
Rescue of camptothecin sensitivity by a single wild-type *Cyp6d2* transgene. All data points represent three to four independent trials of five to six vials each. Error bars represent SDs. (A−B) Rescue of both *cyp6d2* mutants. A precise excision of *P{GT1}CG42565* was used as the *cyp6d2*^NT^ mutant in (B). (C) Partial rescue of the *brca2^47^* allele.

We crossed the *attP-Cyp6d2* transgenic stock to our *cyp6d2*^SD^ mutant flies to generate flies that carried both the mutation and the rescue construct and conducted sensitivity assays with these flies. *cyp6d2*^SD^ homozygotes carrying one copy of the rescue construct showed nearly 100% survival at all doses tested (Figure 4A). We conclude that *cyp6d2*^SD^ is the *scpt* mutation.

### A second, naturally occurring Cyp6d2 variant also sensitizes flies to camptothecin

During the course of examining *Cyp6d2* sequences in other fly stocks, we discovered a second set of polymorphisms that produced a nearly identical camptothecin sensitivity phenotype (Figure 4B and Figure S2). In these stocks, consecutive asparagine residues (N438 and N439) were changed to aspartic acid and threonine respectively. Interestingly, semiquantitative RT-PCR revealed that this mutant produced little to no *Cyp6d2* transcript (Figure 3, C−E). We named this mutant *cyp6d2*^NT^ (no transcript). Sequence analysis showed that *Mi{ET1}* and *P{GT1}* insertion stocks, as well as DrosDel collection deficiencies carried the *cyp6d2*^NT^ mutation (Table 1). The mutant phenotype of these flies was also completely rescued by the wild-type construct (Figure 4B).

We developed allele-specific primers to rapidly screen additional stocks for either of the *cyp6d2* mutations (Figure S1; see *Materials and Methods*). For the *cyp6d2*^NT^ allele, we used allele-specific primers specific to the N438D and N439T polymorphisms. Table 1 lists all stocks containing transposable elements and deficiencies that we have examined via complementation tests and/or PCR. Notably, multiple stocks with transposable elements or deficiencies on the X and 3rd chromosomes also were shown to carry the point mutations in *Cyp6d2*.

### The *brca2^47^* phenotype results from defects in both HR and Cyp6d2

We reasoned that the *brca2*^47^ mutant could be especially sensitive to camptothecin for two reasons: (1) it is unable to repair DSBs by HR and (2) it carries the *cyp6d2*^SD^ mutation. To test this, we attempted to rescue the sensitivity phenotype of the *brca2*^47^ mutant with the wild-type *Cyp6d2* construct. Importantly, the construct provided only a partial rescue of the sensitivity (Figure 4C). Interestingly, the sensitivity of the Cyp6d2-rescued *brca2*^47^ mutants is similar to the *brca2*^KO^ stock (Figure 1A). These findings demonstrate that the *brca2*^47^ mutant phenotype results from two mutations and strongly suggest that the rescue of the *cyp6d2*^SD^ and *cyp6d2*^NT^ mutant phenotypes by a wild-type copy of *Cyp6d2* is not simply the result of overexpressing a detoxification gene.

## Discussion

In this study, we have shown that many Drosophila stocks carry mutations in *Cyp6d2* that render the flies hypersensitive to the drug camptothecin. We hypothesize that Cyp6d2 is a critical enzyme required for the breakdown and/or removal of camptothecin. In its absence, camptothecin levels remain high and cause significant cell death, most likely through the creation of one-ended DSBs. When combined with a mutation in the DSB repair gene, *brca2*, we observed a synergistic effect. Therefore, we hypothesize that at low doses of camptothecin, the resulting DSBs can be repaired through Brca2-dependent HR (Figure 1A).

These variants of *Cyp6d2* illustrate a potential pitfall in the study of DNA repair genes in Drosophila. It is likely that many genes involved in detoxification or removal of harmful compounds are polymorphic. Such polymorphisms may skew drug sensitivity screens such that the results are misinterpreted. In an attempt to understand the role of HR proteins in one-ended DSB repair, we used camptothecin sensitivity assays to characterize different DNA repair mutants. We initially were misled to believe that *brca2* mutants were exceptionally sensitive to camptothecin, more so than *rad51* mutants (data not shown), suggesting a function for Drosophila Brca2 in camptothecin-induced damage repair outside of its well-defined role in HR. However, our results here, along with the observation that *rad51* and *brca2* mutants display similar sensitivities to topotecan (data not shown), suggest that this is not the case.

### Cytochrome P450s and detoxification of xenobiotics

The cytochrome P450 superfamily, of which Drosophila *Cyp6d2* is a member, is a group of metabolic enzymes with an unusually wide range of substrates (Coon *et al.* 1992). They are conserved throughout evolution (Coon *et al.* 1996) and are involved in the metabolism of many endogenous and exogenous compounds. P450 enzymes catalyze the addition of oxygen to a substrate via a heme cofactor (Coon *et al.* 1992; Hollenberg 1992). The additional oxygen atom may alter the stability of the substrate, leading to other molecular rearrangements (Coon *et al.* 1996; Bergé *et al.* 1998), or it may trigger conjugation by enzymes such as glutathione-S-transferases (reviewed in Tu and Akgül 2005). These processes lead to the detoxification and/or excretion of harmful compounds.

P450 activity is critically dependent on the heme ligand, which mediates the electron transfer reactions that ultimately lead to a modified substrate (Hollenberg 1992). The heme group is bound to the protein via a cysteine residue near its C-terminus and surrounded by conserved sequence (Tijet *et al.* 2001). Notably, the *cyp6d2*^SD^ mutation is located very close to the heme-binding cysteine in Cyp6d2 (Figure 3B). In addition to altering the C-terminus of the protein sequence, this mutation also results in structural changes very close to the heme-binding cysteine. Because the *cyp6d2*^SD^ mutant appears slightly more resistant to camptothecin than the *cyp6d2*^NT^ mutant (Figure 4, A and B), we hypothesize that protein encoded by the *cyp6d2*^SD^ allele has greatly reduced function and is a severe hypomorph.

The amino acid changes in *cyp6d2*^NT^ must be closely linked to a noncoding change that affects the regulation of *Cyp6d2*. Because the camptothecin sensitivity in *cyp6d2*^NT^ was successfully rescued by the transgene, such a change would have to be within the region contained in the rescue construct. Although there are no obvious changes in the promoter sequence that would be predicted to affect transcription initiation, the 3′ UTR of *cyp6d2*^NT^ is significantly different from wild-type sequence (Figure S2).

### Potential roles of Drosophila Cyp6d2

In Drosophila, chemical detoxification occurs in the Malpighian tubules, midgut and larval fat body. Consistent with a role in detoxification, the organ with the highest level of expression of *Cyp6d2* is the larval fat body (Chintapalli *et al.* 2007; Yang *et al.* 2007). Temporally, the greatest expression levels of Cyp6d2 occur during the larval and prepupal stages (Chintapalli *et al.* 2007), which coincide with the days immediately after treatment in our sensitivity assays. *Cyp6d2* expression was very high in hyperoxic conditions (Gruenewald *et al.* 2009), but it was not identified in a survey of multiple xenobiotic and insecticide screens (Giraudo *et al.* 2010). Therefore, its preferred substrates are unknown.

There are 83 functional P450 genes in the Drosophila genome, including 22 from the insect-specific *CYP6* family (Tijet *et al.* 2001). Considering that P450 enzymes frequently have overlapping substrate specificity (Coon *et al.* 1992; Hollenberg 1992), it is reasonable to expect that another P450 enzyme could detoxify camptothecin in the absence of Cyp6d2. However, our data suggest that Cyp6d2 provides a nonredundant function in camptothecin detoxification. It is possible that camptothecin removal or breakdown could also involve multiple steps, with additional enzymes mediating other reactions. In this case, at least one of the reactions must require Cyp6d2.

The fact that *scpt* mutants are sensitive to camptothecin but not topotecan was an unexpected result. We propose three hypotheses to explain this. First, camptothecin may have alternate cytotoxicity separate from its inhibition of Top1. The initial discovery and chemical characterization of camptothecin was promising, but clinical trials were abandoned after issues of toxicity arose (Wall and Wani 1995; Legarza and Yang 2006). Later work prompted the development of topotecan and irinotecan (Kunimoto *et al.* 1987; Mattern *et al.* 1991). The camptothecin sensitivity we observed in fruit flies could be caused by the same mechanisms that resulted in the side effects observed in human clinical trials. This would suggest that Cyp6d2-mediated breakdown or removal of camptothecin prevents this toxicity from reaching lethal levels in flies.

Alternatively, a second hypothesis is that the active site of Cyp6d2 can accommodate camptothecin but not topotecan. The most likely explanation for such specificity would be the chemical properties of the two drugs. Camptothecin is a lipophilic molecule, whereas topotecan is hydrophilic. The human P450 enzymes most critical for drug detoxification, including Cyp3a4, tend to favor lipophilic substrates (Smith *et al.* 1997). Despite being structurally similar, the water-soluble topotecan (Pommier 2009) may not be a suitable substrate for Cyp6d2, which would explain why *cyp6d2* mutants are not sensitive to the drug.

Our third hypothesis posits that lipophilic camptothecin may accumulate in the fat body (Zijlstra and Vogel 1988), which functions analogously to the liver in fruit flies and other insects (Buchon *et al.* 2009; Yang *et al.* 2007). In contrast, topotecan, being hydrophilic, may be more easily excreted, resulting in a shorter time of exposure to the drug. The observation that Cyp6d2 is highly expressed in the fat body (Yang *et al.* 2007), supports this model.

All of these hypotheses assume that Cyp6d2 is involved in camptothecin detoxification, but other possible explanations may exist. Recently, Cyp6d2 was shown to be upregulated 18 hr after ionizing radiation treatment, independent of p53 (van Bergeijk *et al.* 2012). This suggests that Cyp6d2 may have a role in p53-independent apoptosis. A defective apoptotic pathway could impair imaginal disc development in larvae, leading to the adult lethality that we observe.

Drosophila provides an excellent *in vivo* system to study the effects of DNA repair mutations and mutagens on genome stability and survival. Nevertheless, our studies suggest caution when using Drosophila as a tool for drug screening. Care must be taken in any analysis of phenotypes because of the possibility that genetic background effects, such as the *cyp6d2* mutations, could be influencing the results.

Our study also has implications for the development of cancer chemotherapeutics. P450 enzymes and the polymorphisms that exist within them pose significant challenges in drug development (Guengerich 1999; Plant 2007; Maekawa *et al.* 2010). Point mutations in Drosophila P450 genes have previously been shown to dramatically affect drug detoxification (Amichot *et al.* 2004), suggesting fruit flies may be a valuable model for this research. Because many cancer chemotherapeutics damage DNA or target defective DNA repair mechanisms (Ferguson and Pearson 1996; Lawley and Phillips 1996), studying these drugs in Drosophila allows us to evaluate the influences of both detoxification and repair enzymes simultaneously.

## Supplementary Material

Supporting Information
