# Supplementary material for: Common Variants of Drosophila melanogaster Cyp6d2 Cause Camptothecin Sensitivity and Synergize With Loss of Brca2
Source: G3 (Bethesda). 2013 Jan 1;3(1):91–9. doi: 10.1534/g3.112.003996 (PMC3538347; doi:10.1534/g3.112.003996)
Supplement: Supporting Information [file supp_3_1_91__index.html]

Supporting Information 

# Common Variants of *Drosophila melanogaster* Cyp6d2 Cause Camptothecin Sensitivity and Synergize With Loss of Brca2

## Supporting Information for Thomas *et al.*, 2013

**Files in this Data Supplement:**

- Supporting Information - Figures S1 and S2 and Table S1 (PDF, 322 KB)
- Figure S1 - Sample allele-specific PCR gel (PDF, 304 KB)
- Figure S2 - Nucleotide sequence of *Cyp6d2* alleles (PDF, 80 KB)
- Table S1 - Amino acid changes found in non-complementing region (PDF, 54 KB)
