# Supplementary material for: Common Variants of Drosophila melanogaster Cyp6d2 Cause Camptothecin Sensitivity and Synergize With Loss of Brca2
Source: G3 (Bethesda). 2013 Jan 1;3(1):91–9. doi: 10.1534/g3.112.003996 (PMC3538347; doi:10.1534/g3.112.003996)
Supplement: Supporting Information [file supp_3.1.91_003996SI.pdf]

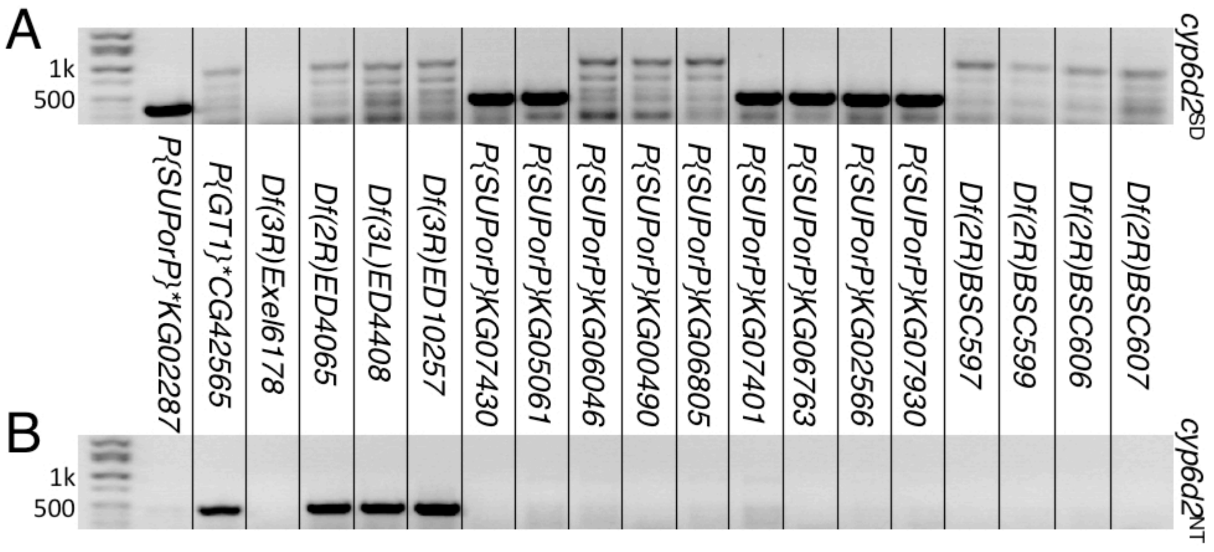

Figure S1 Sample allele-specific PCR gel. (*) indicates a precise excision of the insertion shown. Both PCR products are around 400 bp (see Materials and Methods). Sizes of molecular weight markers are indicated. (A) Allele-specific PCR gel for *cyp6d2*^{SD} mutation. *P{SUPorP}*KG02287* serves as a positive control. (B) Allele-specific PCR gel for *cyp6d2*^{NT} mutation. *P{GT1}*CG42565* serves as a positive control.

```
WT    -277     AAGAACCACATTCTATTTGGGGTCAACATATTGAAAGCATAATGATATGCTGTTGATATC
SD             AAGAACCACATTCTATTTGGGGTCAACATATTGAAAGCATAATGATATGCTGTTGATATC
NT             AAGAACCACATTCTATTTGGGGTCAACATATTGAAAGCATAATGATATGCTGTTGAAATC
               ******************************************************** ***

WT    -217     ATATACCTTTGAAATTAGTTGTGCATTTCTGTAATGTGCTCAACAGGTAGTCTCTTTGCT
SD             ATATACCTTTGAAATTAGTTGTGCATTTCTGTAATGTGCTCAACAGGTAGTCTCTTTGCT
NT             ATATACCTTTGAAATTAGTTGTGCATTTCTGTCATGGGCTCAACAGGTAGTCTCTTTGCT
               ******************************** *** ***********************

WT    -157     TTTTGCCGCCCTGCTTCCCCTGAAGAGTCGAAAGCTTTTTCCAGCTGAGAAGTCACTTGT
SD             TTTTGCCGCCCTGCT--CCCTGAAGAGTCGAAAGCTTTTTCCAGCTGAGAAGTCACTTGT
NT             TTTTGCCGCCCTGCTTCCCCTGAAGAGTCGAAAGCTTTTTCCAGCTGAGAAGTCACTTGT
               ***************  *******************************************

WT     -97     TGATTTGCTGGAAGAGCAGCAGAGTCCAGAGAAAAAGACTCGGTTGTTTGTAGCGCTTAT
SD             TGATTTGCTGGAAGAGCAGCAGAGTCCAGAGAAAAAGACTCGGTTGTTTGTAGCGCTTAT
NT             TGATTTGCTGGAAGAGCAGCAGAGTCCAGAGAAAAAGACTCGGTTGTTTGTAGCGCTTAT
               ************************************************************

WT     -37     CGCGGGTTCTACGTCTATATAAGACCGATGCTCATGCAGTCGAATTGTACAGTTTGGAAG
SD             CGCGGGTTCTACGTCTATATAAGACCGATGCTCATGCAGTCGAATTGTACAGTTTGGAAG
NT             CGCGGGTTCTACGTCTATATAAGACCGATGCTCATGCAGTCGAATTGTACAGTTTGGAAG
               ************************************************************

WT      24     GTGTGCATCGAGTAGTTCGCAAATATTTCACCACTTTTAGAAATTGTCGATTTTCAATAC
SD             GTGTGCATCGAGTAGTTCGCAAATATTTCACCACTTTTAGAAATTGTCGATTTTCAATAC
NT             GTGTGCATCGAGTAGTTCGCAAATATTTCACCACTTTTAGAAATTGTCGATTTTCAATAC
               ************************************************************

WT      84     CAAAAGTTAGACTAGAGATTCTCAATATGTGGACCATTCTCCTGACGATCCTAATCGCGG
SD             CAAAAGTTAGACTAGAGATTCTCGATATGTGGACCATTCTCCTGACGATCCTAATCGCGG
NT             CAAAAGTTAGACTAGAGATTCTCAATATGTGGACCATTCTCCTGACGATCCTAATCGCGG
               *********************** ************************************

WT     144     GTCTGCTGTACAGATATGTGAAGCGGCACTACACTCACTGGCAGCGATTGGGAGTCGATG
SD             GTCTGCTGTACAGATATGTGAAGCGGCACTACACTCACTGGCAGCGATTGGGAGTCGATG
NT             GTCTGCTGTACAGATATGTGAAGCGGCACTACACTCACTGGCAGCGATTGGGAGTCGATG
               ************************************************************

WT     204     AGGAGCCGGCAAAGATTCCCTTTGGCGTAATGGATACTGTCATGAAGCAGGAACGAAGTT
SD             AGGAGCCGGCAAAGATTCCCTTTGGCGTAATGGATACTGTCATGAAGCAGGAACGAAGTT
NT             AGGAGCCGGCAAAGATTCCCTTTGGCGTAATGGATACTGTCATGAAGCAGGAACGAAGTT
               ************************************************************

WT     264     TGGGCATGGCTCTGGCTGATATATACGCCCGGCACGAGGGCAAAATCGTTGGCATATACA
SD             TGGGCATGGCTCTGGCTGATATATACGCCCGGCACGAGGGCAAAATCGTTGGCATATACA
NT             TGGGCATGGCTCTGGCTGATATATACGCCCGGCACGAGGGCAAAATCGTTGGCATATACA
               ************************************************************

WT     324     TGTTGAACAAACGAAGTATCCTGATCCGAGATGCCCAATTGGCTCGCCAAATCATGACCA
SD             TGTTGAACAAACGAAGTATCCTGATCCGAGATGCCCAATTGGCTCGCCAAATCATGACCA
NT             TGTTGAACAAACGAAGTATCCTGATCCGAGATGCCCAATTGGCTCGCCAAATCATGACCA
               ************************************************************

WT     384     GCGATTTCGCCAGTTTCCACGATCGCGGGGTGTACGTGGACGAGGATAAGGACCCGTTGT
SD             GCGATTTCGCCAGTTTCCACGATCGCGGGGTGTACGTGGACGAGGATAAGGACCCGTTGT
NT             GCGATTTCGCCAGTTTCCACGATCGCGGGGTGTACGTGGACGAGGATAAGGACCCGTTGT
               ************************************************************

WT     444     CGGCCAACCTTTTTAACCTAAGAGGTGCTTCATGGCGAAATCTACGTCAGAAACTTACGC
SD             CGGCCAACCTTTTTAACCTAAGAGGTGCTTCATGGCGAAATCTACGTCAGAAACTTACGC
NT             CGGCCAACCTTTTTAACCTAAGAGGTGCTTCATGGCGAAATCTACGTCAGAAACTTACGC
               ************************************************************
```

```
WT      504     CGTCGTTCTCCTCCGGCAAAATTAAGGGCATGTTCGGCACCATCGACGACGTGGGCGATA
SD              CGTCGTTCTCCTCCGGCAAAATTAAGGGCATGTTCGGCACCATCGACGACGTGGGCGATA
NT              CGTCGTTCTCCTCCGGCAAAATTAAGGGCATGTTCGGCACCATCGACGACGTGGGCGATA
                ************************************************************

WT      564     AGTTGGTGCAGCATTTGGAGGGGGCCCTTGACCAAAGCGATGAAGTGGAGATCAAGGATG
SD              AGTTGGTGCAGCATTTGGAGGGGGCCCTTGACCAAAGCGATGAAGTGGAGATCAAGGATG
NT              AGTTGGTGCAGCATTTGGAGGGGGCCCTTGACCAAAGCGATGAAGTGGAGATCAAGGATG
                ************************************************************

WT      624     TGATGACCACGTATGCGGTGGACATCATTGGGTCAGTCATTTTTGGCTTGGAGATAGACA
SD              TGATGACCACGTATGCGGTGGACATCATTGGGTCAGTCATTTTTGGCTTGGAGATAGACA
NT              TGATGACCACGTATGCGGTGGACATCATTGGGTCAGTCATTTTTGGCTTGGAGATAGACA
                ************************************************************

WT      684     GCTTCCGAAATCCAAAAAACGAGTTCCGCGAGATCAGCAGCTCTACGTCTAGGGATGAAT
SD              GCTTCCGAAATCCAAAAAACGAGTTCCGCGAGATCAGCAGCTCTACGTCTAGGGATGAAT
NT              GCTTCCGAAATCCAAAAAACGAGTTCCGCGAGATCAGCAGCTCTACGTCTAGGGATGAAT
                ************************************************************

WT      744     CTCTGCTGCTGAAGATTCACAATATGTCCATGTTCATTTGTCCACCgtgagttcaaaaat
SD              CTCTGCTGCTGAAGATTCACAATATGTCCATGTTCATTTGTCCACCgtgagttcaaaaat
NT              CTCTGCTGCTGAAGATTCACAATATGTCCATGTTCATTTGTCCACCgtgagttcaaaaat
                ************************************************************

WT      804     aacccagggtatatatttgggaatctttatttctatatttataactaaattttgtacagG
SD              aacccagggtatatatttgggaatctttatttctatatttataactaaattttgtacagG
NT              aacccagggtatatatttgggaatctttatttctatatttataactaaattttgtacagG
                ************************************************************

WT      864     ATTGCTAAGCTAATGAACCGCTTGGGCTACGAAAGCCGCATTCTGACATCCCTGCGAGAC
SD              ATTGCTAAGCTAATGAACCGCTTGGGCTACGAAAGCCGCATTCTGACATCCCTGCGAGAC
NT              ATTGCTAAGCTAATGAACCGCTTGGGCTACGAAAGCCGCATTCTGACATCCCTGCGAGAC
                ************************************************************

WT      924     ATGATGAAGCGCACAATCGAGTTCCGGGAGGAGCACAACGTGGTGCGAAAGGACATGCTG
SD              ATGATGAAGCGCACAATCGAGTTCCGGGAGGAGCACAACGTGGTGCGAAAGGACATGCTG
NT              ATGATGAAGCGCACAATCGAGTTCCGGGAGGAGCACAACGTGGTGCGAAAGGACATGCTG
                ************************************************************

WT      984     CAGCTGCTGATCCGCCTCCGAAATACGGGCAAGATTGGCGAGGACGACGACCAGGTGTGG
SD              CAGCTGCTGATCCGCCTCCGAAATACGGGCAAGATTGGCGAGGACGACGACCAGGTGTGG
NT              CAGCTGCTGATCCGCCTCCGAAATACGGGCAAGATTGGCGAGGACGACGACCAGGTGTGG
                ************************************************************

WT     1044     GACATGGAAACCGCACAGGAGCAGCTGAAGTCTATGTCGATCGAGAAGATTGCCGCCCAG
SD              GACATGGAAACCGCACAGGAGCAGCTGAAGTCTATGTCGATCGAGAAGATTGCCGCCCAG
NT              GACATGGAAACCGCACAGGAGCAGCTGAAGTCTATGTCGATCGAGAAGATTGCCGCCCAG
                ************************************************************

WT     1104     GCATTCCTGTTCTACGTGGCGGGCTCGGAATCCACAGCTGCGGCGTCTGCCTTCACTCTC
SD              GCATTCCTGTTCTACGTGGCGGGCTCGGAATCCACAGCTGCGGCGTCTGCCTTCACTCTC
NT              GCATTCCTGTTCTACGTGGCGGGCTCGGAATCCACAGCTGCGGCGTCTGCCTTCACTCTC
                ************************************************************

WT     1164     TACGAGCTGTCCATGTATCCGGAACTGCTGAAGGAGGCGCAAGAAGAAGTGGATGCTGTG
SD              TACGAGCTGTCCATGTATCCGGAACTGCTGAAGGAGGCGCAAGAAGAAGTGGATGCTGTG
NT              TACGAGCTGTCCATGTATCCGGAACTGCTGAAGGAGGCGCAAGAAGAAGTGGATGCTGTG
                ************************************************************

WT     1224     CTGATGAAGCACAATCTGAAGCCCAAGGATAGGTTCACCTACGAGGCGGTGCAGGACTTA
SD              CTGATGAAGCACAATCTGAAGCCCAAGGATAGGTTCACCTACGAGGCGGTGCAGGACTTA
NT              CTGATGAAGCACAATCTGAAGCCCAAGGATAGGTTCACCTACGAGGCGGTGCAGGACTTA
```

```
                        ************************************************************

WT     1284     AAGTTTCTGGACATCTGCATCATGGgtgggtttcacatagcattcagtttcaacgatcct
SD              AAGTTTCTGGACATCTGCATCATGGgtgggtttcacatagcattcagtttcaacgatcct
NT              AAGTTTCTGGACATCTGCATCATGGgtgggtttcacatagcattcagttttaacgatcct
                ************************************************** *********

WT     1344     tagttaaacgtatttttgcagAAACCATTCGCAAATACCCCGGGCTACCATTCCTCAATC
SD              tagttaaacgtatttttgcagAAACCATTCGCAAATACCCCGGGCTACCATTCCTCAATC
NT              tagttaaacgtatatttgcagAAACCATTCGCAAATACCCCGGGCTACCATTCCTCAATC
                ************* **********************************************

WT     1404     GAGAGTGCACCGAAGATTATCCCGTACCTGGAACTAATCACATCATAGCCAAGGGAACAC
SD              GAGAGTGCACCGAAGATTATCCCGTACCTGGAACTAATCACATCATAGCCAAGGGAACAC
NT              GAGAGTGCACCGAAGATTATCCCGTACCTGGAACTAATCACATCATAGCCAAGGGAACAC
                ************************************************************

WT     1464     CCATTCTGATCTCGCTCTTTGGCATGCAGCGTGATCCCGTCTATTTTCCCAATCCCAATG
SD              CCATTCTGATCTCGCTCTTTGGCATGCAGCGTGATCCCGTCTATTTTCCCAATCCCAATG
NT              CCATTCTGATCTCGCTCTTTGGCATGCAGCGTGATCCCGTCTATTTTCCCAATCCCAATG
                ************************************************************

WT     1524     GCTACGATCCCCATCGCTTCGATTCAAATAACATGAACTACGATCAGGCAGCTTATATGC
SD              GCTACGATCCCCATCGCTTCGATTCAAATAACATGAACTACGATCAGGCAGCTTATATGC
NT              GCTACGATCCCCATCGCTTCGATTCAGATACCATGAACTACGATCAGGCAGCTTATATGC
                ************************** *** *****************************

WT     1584     CTTTTGGAGAGGGTCCTAGGCACTGCATAGgtaaggatgtgttttgtaccagtggtcttg
SD              CTTTTGGAGAGGGTCCTAGGCACTGCATACgtaaagatgtgttttgtaccagtggtcttg
NT              CCTTTGGAGAGGGTCCTAGGCACTGCATAGgtaaggatgtgttttgtaccagtggtcttg
                * *************************** **** *************************

WT     1644     tcttcttcctgatcctgttctcttaattctttgccccaagCTCTTCGCATGGGAAAAGTC
SD              tcttcttcctgatcctgttctcttaattctttgccccaagCTCTTCGCATGGGAAAAGTC
NT              tcttcttcctgatcctgttctcttaattcttttccccaagCTCTTCGCATGGGAAAAGTC
                ******************************** ***************************

WT     1704     AACTCCAAGGTGGCGGTGGCTAAGATTTTGGCCAATTTTGATCTGGTTCAATCTCCACGC
SD              AACTCCAAGGTGGCGGTGGCTAAGATTTTGGCCAATTTTGATCTGGTTCAATCTCCACGC
NT              AACTCCAAGGTGGCGGTGGCTAAGATTTTGGCCAATTTTGATCTGGTTCAATCTCCACGC
                ************************************************************

WT     1764     AAGGAGGTGGAGTTCCGTTTCGATGCCGCCCCTGTTTTAGTGACCAAGGAGCCGTTGAAG
SD              AAGGAGGTGGAGTTCCGTTTCGATGCCGCCCCTGTTTTAGTGACCAAGGAGCCGTTGAAG
NT              AAGGAGGTGGAGTTCCGTTTCGATGCCGCCCCTGTTTTAGTGACCAAGGAGCCGTTGAAG
                ************************************************************

WT     1824     CTGCGCTTGACCAAGAGAAAGTAAATCAAAAATATGTGGCTATTGTATG--------TAA
SD              CTGCGCTTGACCAAGAGAAAGTAAATCAAAAATATGTGGCTATTGTATG--------TAA
NT              CTGCGCTTGACCAAGAGAAAGTAAATCAAAAATATGTGGCTAAAAGCGAATATACTATAT
                ******************************************               **

WT     1876     TTGCAATTTGTATGTATAGATATATATCGATACTCAAATAAATTAATACATTGGTTTTAT
SD              TTGCAATTTGTATGTATAGATATATATCGATACTCAAATAAATTAATACATTGGTTTTAT
NT              AATAAATTTTTATGTATAGATATATATCGATACTCAAATAAATTAATACATTGGTTTTAT
                    ***** **************************************************

WT     1936     TGATCATCTACCAAGATAAT
SD              TGATCATCTACCAAGATAAT
NT              TGATCATCTACCAAGATAAT
                ********************
```

Figure S2 Nucleotide sequence of *Cyp6d2* alleles. Nucleotide positions relative to *Cyp6d2* transcription start site are indicated. Red: untranslated regions, Blue: protein coding regions, Black uppercase: upstream intergenic region, Black lowercase: introns. Asterisk below sequence indicates all three alleles are identical at this position. Highlighted SNPs predicted to change amino acid sequence. Two stocks for each allele were sequenced to confirm changes: WT (*P{SUPor-P}KG06046*, *P{SUPor-P}KG01596*), SD (*P{SUPor-P}KG02287*, *P{SUPor-P}KG06675*), NT (*Mi{ET1}CG3746*, *P{GT1}CG42565*). Alignment performed using Clustal Omega (Sievers et al. 2011) and boundaries are as defined by FlyBase.

Sievers F, Wilm A, Dineen D, Gibson TJ, Karplus K, Li W, Lopez R, McWilliam H, Remmert M, ding JSO, et al. 2011. Fast, scalable generation of high-quality protein multiple sequence alignments using Clustal Omega. *Molecular Systems Biology* **7**: 1–6.

Table S1   Amino acid changes found in non-complementing region

Gene	Mutation
CG30195	I12S
CG34445	D26V
	E55K
	K128Q
CG3746	E20G
	S44A
Cyp6d2	A459P/R
CG13511	A49T
