# Supplementary material for: Common Variants of Drosophila melanogaster Cyp6d2 Cause Camptothecin Sensitivity and Synergize With Loss of Brca2
Source: G3 (Bethesda). 2013 Jan 1;3(1):91–9. doi: 10.1534/g3.112.003996 (PMC3538347; doi:10.1534/g3.112.003996)
Supplement: Supporting Information [file supp_3.1.91_FigureS1.pdf]

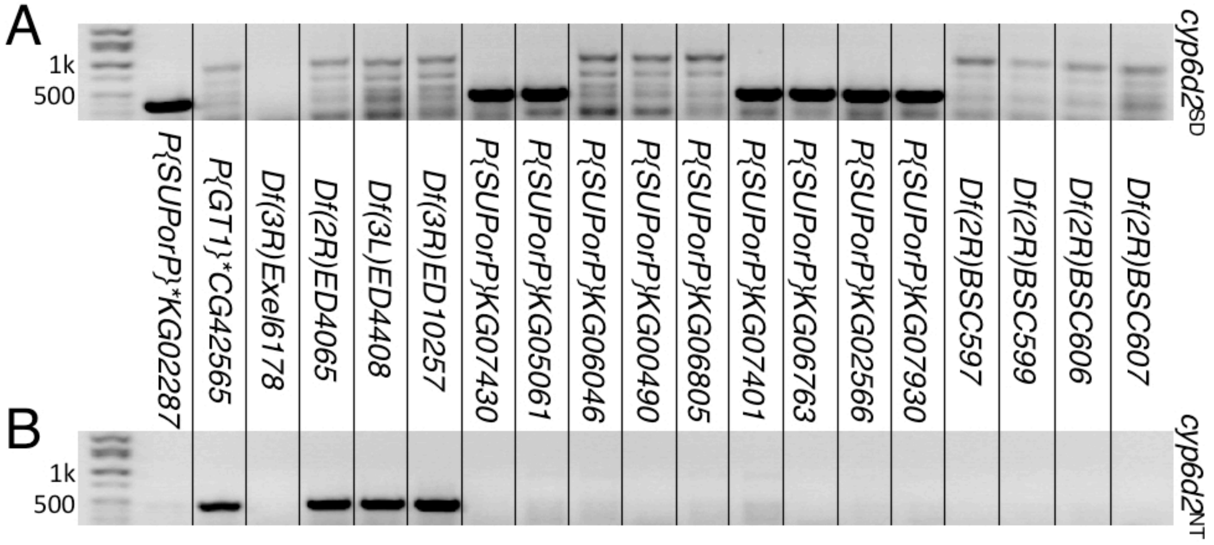

Figure S1 Sample allele-specific PCR gel. (*) indicates a precise excision of the insertion shown. Both PCR products are around 400 bp (see Materials and Methods). Sizes of molecular weight markers are indicated. (A) Allele-specific PCR gel for *cyp6d2*^{SD} mutation. *P{SUPorP}*KG02287* serves as a positive control. (B) Allele-specific PCR gel for *cyp6d2*^{NT} mutation. *P{GT1}*CG42565* serves as a positive control.
