# Supplementary material for: Common Variants of Drosophila melanogaster Cyp6d2 Cause Camptothecin Sensitivity and Synergize With Loss of Brca2
Source: G3 (Bethesda). 2013 Jan 1;3(1):91–9. doi: 10.1534/g3.112.003996 (PMC3538347; doi:10.1534/g3.112.003996)
Supplement: Supporting Information [file supp_3.1.91_FigureS2.pdf]

```
WT    -277      AAGAACCACATTCTATTTGGGGTCAACATATTGAAAGCATAATGATATGCTGTTGATATC
SD              AAGAACCACATTCTATTTGGGGTCAACATATTGAAAGCATAATGATATGCTGTTGATATC
NT              AAGAACCACATTCTATTTGGGGTCAACATATTGAAAGCATAATGATATGCTGTTGAAATC
                ******************************************************** ***

WT    -217      ATATACCTTTGAAATTAGTTGTGCATTTCTGTAATGTGCTCAACAGGTAGTCTCTTTGCT
SD              ATATACCTTTGAAATTAGTTGTGCATTTCTGTAATGTGCTCAACAGGTAGTCTCTTTGCT
NT              ATATACCTTTGAAATTAGTTGTGCATTTCTGTCATGGGCTCAACAGGTAGTCTCTTTGCT
                ******************************** *** ***********************

WT    -157      TTTTGCCGCCCTGCTTCCCCTGAAGAGTCGAAAGCTTTTTCCAGCTGAGAAGTCACTTGT
SD              TTTTGCCGCCCTGCT--CCCTGAAGAGTCGAAAGCTTTTTCCAGCTGAGAAGTCACTTGT
NT              TTTTGCCGCCCTGCTTCCCCTGAAGAGTCGAAAGCTTTTTCCAGCTGAGAAGTCACTTGT
                ***************  *******************************************

WT     -97      TGATTTGCTGGAAGAGCAGCAGAGTCCAGAGAAAAAGACTCGGTTGTTTGTAGCGCTTAT
SD              TGATTTGCTGGAAGAGCAGCAGAGTCCAGAGAAAAAGACTCGGTTGTTTGTAGCGCTTAT
NT              TGATTTGCTGGAAGAGCAGCAGAGTCCAGAGAAAAAGACTCGGTTGTTTGTAGCGCTTAT
                ************************************************************

WT     -37      CGCGGGTTCTACGTCTATATAAGACCGATGCTCATGCAGTCGAATTGTACAGTTTGGAAG
SD              CGCGGGTTCTACGTCTATATAAGACCGATGCTCATGCAGTCGAATTGTACAGTTTGGAAG
NT              CGCGGGTTCTACGTCTATATAAGACCGATGCTCATGCAGTCGAATTGTACAGTTTGGAAG
                ************************************************************

WT      24      GTGTGCATCGAGTAGTTCGCAAATATTTCACCACTTTTAGAAATTGTCGATTTTCAATAC
SD              GTGTGCATCGAGTAGTTCGCAAATATTTCACCACTTTTAGAAATTGTCGATTTTCAATAC
NT              GTGTGCATCGAGTAGTTCGCAAATATTTCACCACTTTTAGAAATTGTCGATTTTCAATAC
                ************************************************************

WT      84      CAAAAGTTAGACTAGAGATTCTCAATATGTGGACCATTCTCCTGACGATCCTAATCGCGG
SD              CAAAAGTTAGACTAGAGATTCTCGATATGTGGACCATTCTCCTGACGATCCTAATCGCGG
NT              CAAAAGTTAGACTAGAGATTCTCAATATGTGGACCATTCTCCTGACGATCCTAATCGCGG
                *********************** ************************************

WT     144      GTCTGCTGTACAGATATGTGAAGCGGCACTACACTCACTGGCAGCGATTGGGAGTCGATG
SD              GTCTGCTGTACAGATATGTGAAGCGGCACTACACTCACTGGCAGCGATTGGGAGTCGATG
NT              GTCTGCTGTACAGATATGTGAAGCGGCACTACACTCACTGGCAGCGATTGGGAGTCGATG
                ************************************************************

WT     204      AGGAGCCGGCAAAGATTCCCTTTGGCGTAATGGATACTGTCATGAAGCAGGAACGAAGTT
SD              AGGAGCCGGCAAAGATTCCCTTTGGCGTAATGGATACTGTCATGAAGCAGGAACGAAGTT
NT              AGGAGCCGGCAAAGATTCCCTTTGGCGTAATGGATACTGTCATGAAGCAGGAACGAAGTT
                ************************************************************

WT     264      TGGGCATGGCTCTGGCTGATATATACGCCCGGCACGAGGGCAAAATCGTTGGCATATACA
SD              TGGGCATGGCTCTGGCTGATATATACGCCCGGCACGAGGGCAAAATCGTTGGCATATACA
NT              TGGGCATGGCTCTGGCTGATATATACGCCCGGCACGAGGGCAAAATCGTTGGCATATACA
                ************************************************************

WT     324      TGTTGAACAAACGAAGTATCCTGATCCGAGATGCCCAATTGGCTCGCCAAATCATGACCA
SD              TGTTGAACAAACGAAGTATCCTGATCCGAGATGCCCAATTGGCTCGCCAAATCATGACCA
NT              TGTTGAACAAACGAAGTATCCTGATCCGAGATGCCCAATTGGCTCGCCAAATCATGACCA
                ************************************************************

WT     384      GCGATTTCGCCAGTTTCCACGATCGCGGGGTGTACGTGGACGAGGATAAGGACCCGTTGT
SD              GCGATTTCGCCAGTTTCCACGATCGCGGGGTGTACGTGGACGAGGATAAGGACCCGTTGT
NT              GCGATTTCGCCAGTTTCCACGATCGCGGGGTGTACGTGGACGAGGATAAGGACCCGTTGT
                ************************************************************

WT     444      CGGCCAACCTTTTTAACCTAAGAGGTGCTTCATGGCGAAATCTACGTCAGAAACTTACGC
SD              CGGCCAACCTTTTTAACCTAAGAGGTGCTTCATGGCGAAATCTACGTCAGAAACTTACGC
NT              CGGCCAACCTTTTTAACCTAAGAGGTGCTTCATGGCGAAATCTACGTCAGAAACTTACGC
                ************************************************************
```

```
WT      504     CGTCGTTCTCCTCCGGCAAAATTAAGGGCATGTTCGGCACCATCGACGACGTGGGCGATA
SD              CGTCGTTCTCCTCCGGCAAAATTAAGGGCATGTTCGGCACCATCGACGACGTGGGCGATA
NT              CGTCGTTCTCCTCCGGCAAAATTAAGGGCATGTTCGGCACCATCGACGACGTGGGCGATA
                ************************************************************

WT      564     AGTTGGTGCAGCATTTGGAGGGGGCCCTTGACCAAAGCGATGAAGTGGAGATCAAGGATG
SD              AGTTGGTGCAGCATTTGGAGGGGGCCCTTGACCAAAGCGATGAAGTGGAGATCAAGGATG
NT              AGTTGGTGCAGCATTTGGAGGGGGCCCTTGACCAAAGCGATGAAGTGGAGATCAAGGATG
                ************************************************************

WT      624     TGATGACCACGTATGCGGTGGACATCATTGGGTCAGTCATTTTTGGCTTGGAGATAGACA
SD              TGATGACCACGTATGCGGTGGACATCATTGGGTCAGTCATTTTTGGCTTGGAGATAGACA
NT              TGATGACCACGTATGCGGTGGACATCATTGGGTCAGTCATTTTTGGCTTGGAGATAGACA
                ************************************************************

WT      684     GCTTCCGAAATCCAAAAAACGAGTTCCGCGAGATCAGCAGCTCTACGTCTAGGGATGAAT
SD              GCTTCCGAAATCCAAAAAACGAGTTCCGCGAGATCAGCAGCTCTACGTCTAGGGATGAAT
NT              GCTTCCGAAATCCAAAAAACGAGTTCCGCGAGATCAGCAGCTCTACGTCTAGGGATGAAT
                ************************************************************

WT      744     CTCTGCTGCTGAAGATTCACAATATGTCCATGTTCATTTGTCCACCgtgagttcaaaaat
SD              CTCTGCTGCTGAAGATTCACAATATGTCCATGTTCATTTGTCCACCgtgagttcaaaaat
NT              CTCTGCTGCTGAAGATTCACAATATGTCCATGTTCATTTGTCCACCgtgagttcaaaaat
                ************************************************************

WT      804     aacccagggtatatatttgggaatctttatttctatatttataactaaattttgtacagG
SD              aacccagggtatatatttgggaatctttatttctatatttataactaaattttgtacagG
NT              aacccagggtatatatttgggaatctttatttctatatttataactaaattttgtacagG
                ************************************************************

WT      864     ATTGCTAAGCTAATGAACCGCTTGGGCTACGAAAGCCGCATTCTGACATCCCTGCGAGAC
SD              ATTGCTAAGCTAATGAACCGCTTGGGCTACGAAAGCCGCATTCTGACATCCCTGCGAGAC
NT              ATTGCTAAGCTAATGAACCGCTTGGGCTACGAAAGCCGCATTCTGACATCCCTGCGAGAC
                ************************************************************

WT      924     ATGATGAAGCGCACAATCGAGTTCCGGGAGGAGCACAACGTGGTGCGAAAGGACATGCTG
SD              ATGATGAAGCGCACAATCGAGTTCCGGGAGGAGCACAACGTGGTGCGAAAGGACATGCTG
NT              ATGATGAAGCGCACAATCGAGTTCCGGGAGGAGCACAACGTGGTGCGAAAGGACATGCTG
                ************************************************************

WT      984     CAGCTGCTGATCCGCCTCCGAAATACGGGCAAGATTGGCGAGGACGACGACCAGGTGTGG
SD              CAGCTGCTGATCCGCCTCCGAAATACGGGCAAGATTGGCGAGGACGACGACCAGGTGTGG
NT              CAGCTGCTGATCCGCCTCCGAAATACGGGCAAGATTGGCGAGGACGACGACCAGGTGTGG
                ************************************************************

WT     1044     GACATGGAAACCGCACAGGAGCAGCTGAAGTCTATGTCGATCGAGAAGATTGCCGCCCAG
SD              GACATGGAAACCGCACAGGAGCAGCTGAAGTCTATGTCGATCGAGAAGATTGCCGCCCAG
NT              GACATGGAAACCGCACAGGAGCAGCTGAAGTCTATGTCGATCGAGAAGATTGCCGCCCAG
                ************************************************************

WT     1104     GCATTCCTGTTCTACGTGGCGGGCTCGGAATCCACAGCTGCGGCGTCTGCCTTCACTCTC
SD              GCATTCCTGTTCTACGTGGCGGGCTCGGAATCCACAGCTGCGGCGTCTGCCTTCACTCTC
NT              GCATTCCTGTTCTACGTGGCGGGCTCGGAATCCACAGCTGCGGCGTCTGCCTTCACTCTC
                ************************************************************

WT     1164     TACGAGCTGTCCATGTATCCGGAACTGCTGAAGGAGGCGCAAGAAGAAGTGGATGCTGTG
SD              TACGAGCTGTCCATGTATCCGGAACTGCTGAAGGAGGCGCAAGAAGAAGTGGATGCTGTG
NT              TACGAGCTGTCCATGTATCCGGAACTGCTGAAGGAGGCGCAAGAAGAAGTGGATGCTGTG
                ************************************************************

WT     1224     CTGATGAAGCACAATCTGAAGCCCAAGGATAGGTTCACCTACGAGGCGGTGCAGGACTTA
SD              CTGATGAAGCACAATCTGAAGCCCAAGGATAGGTTCACCTACGAGGCGGTGCAGGACTTA
NT              CTGATGAAGCACAATCTGAAGCCCAAGGATAGGTTCACCTACGAGGCGGTGCAGGACTTA
```

```
                ************************************************************

WT    1284      AAGTTTCTGGACATCTGCATCATGGgtgggtttcacatagcattcagtttcaacgatcct
SD              AAGTTTCTGGACATCTGCATCATGGgtgggtttcacatagcattcagtttcaacgatcct
NT              AAGTTTCTGGACATCTGCATCATGGgtgggtttcacatagcattcagttttaacgatcct
                ************************************************** *********

WT    1344      tagttaaacgtatttttgcagAAACCATTCGCAAATACCCCGGGCTACCATTCCTCAATC
SD              tagttaaacgtatttttgcagAAACCATTCGCAAATACCCCGGGCTACCATTCCTCAATC
NT              tagttaaacgtatatttgcagAAACCATTCGCAAATACCCCGGGCTACCATTCCTCAATC
                ************* **********************************************

WT    1404      GAGAGTGCACCGAAGATTATCCCGTACCTGGAACTAATCACATCATAGCCAAGGGAACAC
SD              GAGAGTGCACCGAAGATTATCCCGTACCTGGAACTAATCACATCATAGCCAAGGGAACAC
NT              GAGAGTGCACCGAAGATTATCCCGTACCTGGAACTAATCACATCATAGCCAAGGGAACAC
                ************************************************************

WT    1464      CCATTCTGATCTCGCTCTTTGGCATGCAGCGTGATCCCGTCTATTTTCCCAATCCCAATG
SD              CCATTCTGATCTCGCTCTTTGGCATGCAGCGTGATCCCGTCTATTTTCCCAATCCCAATG
NT              CCATTCTGATCTCGCTCTTTGGCATGCAGCGTGATCCCGTCTATTTTCCCAATCCCAATG
                ************************************************************

WT    1524      GCTACGATCCCCATCGCTTCGATTCAAATAACATGAACTACGATCAGGCAGCTTATATGC
SD              GCTACGATCCCCATCGCTTCGATTCAAATAACATGAACTACGATCAGGCAGCTTATATGC
NT              GCTACGATCCCCATCGCTTCGATTCAGATACCATGAACTACGATCAGGCAGCTTATATGC
                ************************** *** *****************************

WT    1584      CTTTTGGAGAGGGTCCTAGGCACTGCATAGgtaaggatgtgttttgtaccagtggtcttg
SD              CTTTTGGAGAGGGTCCTAGGCACTGCATACgtaaagatgtgttttgtaccagtggtcttg
NT              CCTTTGGAGAGGGTCCTAGGCACTGCATAGgtaaggatgtgttttgtaccagtggtcttg
                * *************************** **** *************************

WT    1644      tcttcttcctgatcctgttctcttaattctttgccccaagCTCTTCGCATGGGAAAAGTC
SD              tcttcttcctgatcctgttctcttaattctttgccccaagCTCTTCGCATGGGAAAAGTC
NT              tcttcttcctgatcctgttctcttaattcttttccccaagCTCTTCGCATGGGAAAAGTC
                ******************************** ***************************

WT    1704      AACTCCAAGGTGGCGGTGGCTAAGATTTTGGCCAATTTTGATCTGGTTCAATCTCCACGC
SD              AACTCCAAGGTGGCGGTGGCTAAGATTTTGGCCAATTTTGATCTGGTTCAATCTCCACGC
NT              AACTCCAAGGTGGCGGTGGCTAAGATTTTGGCCAATTTTGATCTGGTTCAATCTCCACGC
                ************************************************************

WT    1764      AAGGAGGTGGAGTTCCGTTTCGATGCCGCCCCTGTTTTAGTGACCAAGGAGCCGTTGAAG
SD              AAGGAGGTGGAGTTCCGTTTCGATGCCGCCCCTGTTTTAGTGACCAAGGAGCCGTTGAAG
NT              AAGGAGGTGGAGTTCCGTTTCGATGCCGCCCCTGTTTTAGTGACCAAGGAGCCGTTGAAG
                ************************************************************

WT    1824      CTGCGCTTGACCAAGAGAAAGTAAATCAAAAATATGTGGCTATTGTATG--------TAA
SD              CTGCGCTTGACCAAGAGAAAGTAAATCAAAAATATGTGGCTATTGTATG--------TAA
NT              CTGCGCTTGACCAAGAGAAAGTAAATCAAAAATATGTGGCTAAAAGCGAATATACTATAT
                ******************************************               **

WT    1876      TTGCAATTTGTATGTATAGATATATATCGATACTCAAATAAATTAATACATTGGTTTTAT
SD              TTGCAATTTGTATGTATAGATATATATCGATACTCAAATAAATTAATACATTGGTTTTAT
NT              AATAAATTTTTATGTATAGATATATATCGATACTCAAATAAATTAATACATTGGTTTTAT
                    ***** **************************************************

WT    1936      TGATCATCTACCAAGATAAT
SD              TGATCATCTACCAAGATAAT
NT              TGATCATCTACCAAGATAAT
                ********************
```

Figure S2 Nucleotide sequence of *Cyp6d2* alleles. Nucleotide positions relative to *Cyp6d2* transcription start site are indicated. Red: untranslated regions, Blue: protein coding regions, Black uppercase: upstream intergenic region, Black lowercase: introns. Asterisk below sequence indicates all three alleles are identical at this position. Highlighted SNPs predicted to change amino acid sequence. Two stocks for each allele were sequenced to confirm changes: WT (*P{SUPor-P}KG06046*, *P{SUPor-P}KG01596*), SD (*P{SUPor-P}KG02287*, *P{SUPor-P}KG06675*), NT (*Mi{ET1}CG3746*, *P{GT1}CG42565*). Alignment performed using Clustal Omega (Sievers et al. 2011) and boundaries are as defined by FlyBase.

Sievers F, Wilm A, Dineen D, Gibson TJ, Karplus K, Li W, Lopez R, McWilliam H, Remmert M, ding JSO, et al. 2011. Fast, scalable generation of high-quality protein multiple sequence alignments using Clustal Omega. *Molecular Systems Biology* **7**: 1–6.
