# Supplementary material for: Common Variants of Drosophila melanogaster Cyp6d2 Cause Camptothecin Sensitivity and Synergize With Loss of Brca2
Source: G3 (Bethesda). 2013 Jan 1;3(1):91–9. doi: 10.1534/g3.112.003996 (PMC3538347; doi:10.1534/g3.112.003996)
Supplement: Supporting Information [file supp_3.1.91_TableS1.pdf]

Table S1 Amino acid changes found in non-complementing region

Gene	Mutation
CG30195	I12S
CG34445	D26V
	E55K
	K128Q
CG3746	E20G
	S44A
Cyp6d2	A459P/R
CG13511	A49T
